# Lost in the woods: Forest vegetation, and not topography, most affects the connectivity of mesh radio networks for public safety

**DOI:** 10.1371/journal.pone.0278645

**Published:** 2022-12-07

**Authors:** Eloise G. Zimbelman, Robert F. Keefe

**Affiliations:** Department of Forest, Rangeland and Fire Sciences, University of Idaho, Moscow, Idaho, United States of America; Universidade Federal de Uberlandia, BRAZIL

## Abstract

Real-time data- and location-sharing using mesh networking radios paired with smartphones may improve situational awareness and safety in remote environments lacking communications infrastructure. Despite being increasingly used for wildland fire and public safety applications, there has been little formal evaluation of the network connectivity of these devices. The objectives of this study were to 1) characterize the connectivity of mesh networks in variable forest and topographic conditions; 2) evaluate the abilities of lidar and satellite remote sensing data to predict connectivity; and 3) assess the relative importance of the predictive metrics. A large field experiment was conducted to test the connectivity of a network of one mobile and five stationary goTenna Pro mesh radios on 24 Public Land Survey System sections approximately 260 ha in area in northern Idaho. Dirichlet regression was used to predict connectivity using 1) both lidar- and satellite-derived metrics (LIDSAT); 2) lidar-derived metrics only (LID); and 3) satellite-derived metrics only (SAT). On average the full network was connected only 32.6% of the time (range: 0% to 90.5%) and the mobile goTenna was disconnected from all other devices 18.2% of the time (range: 0% to 44.5%). RMSE for the six connectivity levels ranged from 0.101 to 0.314 for the LIDSAT model, from 0.103 to 0.310 for the LID model, and from 0.121 to 0.313 for the SAT model. Vegetation-related metrics affected connectivity more than topography. Developed models may be used to predict the connectivity of real-time mesh networks over large spatial extents using remote sensing data in order to forecast how well similar networks are expected to perform for wildland firefighting, forestry, and public safety applications. However, safety professionals should be aware of the impacts of vegetation on connectivity.

## Introduction

Use of new mesh radio devices that pair with smartphones using Bluetooth can facilitate global navigation satellite system—radio frequency (GNSS-RF) location sharing among people and equipment in remote environments. In GNSS-RF networks, transponders or mobile devices obtain their coordinates using integrated GNSS chips and send those coordinates to other devices via radio frequency [1]. This type of technology has potential applications in public safety when communications infrastructure is absent by enabling the exchange of geospatial information among wildland firefighters, search and rescue (SAR) personnel, and other emergency responders to improve situational awareness (SA) [1–4]. This technology may be useful for disaster communications in healthcare [5] and for digital safety applications in forestry [6]. Wildland firefighting is a particularly strenuous and hazardous occupation, with 480 fatalities between 1990 and 2016 [7]. Vehicle- and aviation-related incidents, medical events such as strokes and heart attacks, and fire entrapments were among the leading causes of wildland firefighter fatalities from 2001 to 2012 [8] and from 2007 to 2016 [7]. To address this, efforts have been made to increase safety and reduce injuries and fatalities among wildland firefighters [7]. Section 1114 of the 2019 John D. Dingell, Jr. Conservation, Management, and Recreation Act required implementation of a system to monitor the locations of all active wildland fire resources used by Federal Type 1 incident management teams in the United States [9]. As part of the enacted legislation, interagency Dingell Act Resource Tracking (DART) teams were required to conduct pilot projects to evaluate available resource tracking technologies that could be used on Type 1 incidents [10, 11]. One tracking system evaluated by the DART Team was the Team Awareness Kit (TAK) smartphone app paired with goTenna Pro X mesh networking radios (goTenna Inc, Brooklyn, NY, USA), which allows communication in areas lacking cellular service [11]. goTenna Pro mesh radios pair with smartphones using Bluetooth and enable automatic sharing of GNSS coordinates, text messages, points, and shapes within the TAK app via radio frequency [11]. Previous evaluations of these devices have reported on their general performance and potential for increasing SA [1–4, 6] as well as their ability to transmit to a stationary repeater [11]. However, there has not been a comprehensive, replicated study evaluating the connectivity of goTenna Pro mesh radio devices operating as a network in a range of forest vegetation and terrain types in the northern Rocky Mountain region.

In addition to improved SA in wildland firefighting, GNSS-RF positioning, geofencing, mesh networking, wireless sensor networks (WSNs), and human activity recognition have a range of other possible applications in natural resource safety and management [1]. For example, by monitoring the current safety status and location of individuals relative to workplace hazards, real-time positioning has been evaluated as a tool for improving SA and safety on logging operations [6, 12–15]. Additionally, human activity recognition using wearable and mobile device sensors can be used to quantify occupational tasks in forestry [16, 17], which could ultimately improve health and safety as well as enhance precision forestry and fire management. The use of these technologies, together with the increasing availability of remote sensing and big data, represent components of smart forestry and Forestry 4.0 [18–22]. Forestry 4.0 is based on the adoption of Industry 4.0 concepts in forestry, such as the digitalization, automation, and increasing precision throughout the forestry supply chain [18, 19, 21]. Recent advances in GNSS-RF mesh and mobile ad hoc networks (MANETs) have made it possible to share the locations obtained from the GNSS chip in phones with other paired phones and devices. Specifically, the phones are paired with small radios like goTennas via Bluetooth [1]. The radios are then able to transmit information to other phone/radio pairs in the absence of cellular networks, WiFi, or base stations [1, 23–25]. Depending on the particular technology, users may share locations, send texts, and even make voice calls. Many of these technologies also form mesh, or multi-hop, networks, in which each node in the network can relay data to other nodes [24, 25]. Unlike traditional radios, mesh networking allows information to be routed through other nodes to enable communication between users who may not have a direct line-of-sight (LOS) connection [25]. WSNs, which can also utilize mesh networking, consist of radio frequency transceivers together with small, low-cost wireless sensors capable of measuring a variety of physical parameters such as temperature, humidity, pressure, soil, and light intensity [26–28]. By transmitting field measurements in remote areas, WSNs are useful in environmental monitoring and have been deployed to measure forest health and detect wildfires [1, 27].

Mobile and wireless communications systems such as GNSS-RF devices, smartphones, mesh networks, and WSNs are able to function as data- and location-sharing technologies by utilizing antenna systems to transmit and receive signals. The growing number of mobile and wearable devices together with the expansion of the Internet of Things (IoT) in recent years has led to a need to design compact antennas with high data transmission rates and low energy consumption [28, 29]. On-chip antennas have been designed in order to meet the small size, low-power, and low-loss requirements associated with advances in wireless communications [30]. Recent research has evaluated the use of metamaterials and metasurfaces in order to develop on-chip antennas that are high-performance, structurally simple and cost effective [31–34].

A mesh network is considered connected if a path exists between each pair of nodes [35]. One way to evaluate mesh networks is to calculate the connectivity probability, which is the proportion of time the network is connected over a specified observation period [35]. Traditional approaches to studying landscape-scale connectivity of radio networks have relied heavily on computer-intensive methods that simulate routing protocols [24, 36–38], mobility models [35, 36, 39–43], and radio signal propagation models [44–46]. Radio propagation models can be either theoretical or empirical, and many are derived using both analytical and empirical methods [47, 48]. Because they are based on actual measurements, empirical models can account for all the known or unknown environment-related factors that affect radio wave propagation, but they may not be valid in different environments or at different transmission frequencies [47]. There is also a tradeoff in computational complexity between empirical models and more realistic models that account for geography, terrain, or vegetation [44].

Early research on near-ground communication and radio transmission in forested environments demonstrated the ability of empirical radio propagation models that account for foliage to predict very high frequency (VHF) and ultra high frequency (UHF) path loss in vegetation [49, 50]. More recently, WSNs, which rely on near-ground peer-to-peer propagation and share similar features with MANETs used for public safety, have been evaluated for environmental monitoring and communication in remote vegetated environments [45, 51–55]. Olasupo and Otero proposed a variety of path loss models based on WSN nodes deployed in jungle environments and compared these to theoretical models, most of which were found to under-predict path loss [56]. To move beyond empirical predictions that use only distance and frequency to estimate path loss, Azevedo and Santos developed empirical models that accounted for forest stand parameters including tree density, tree diameter, canopy diameter, and foliage density [57, 58]. Anastassiu et al. developed computational models based on tree geometry and the electrical characteristics of air, soil, and vegetation [53].

In order to address the fact that most simulations evaluating wireless networks assume propagation along a flat plane, Kotz et al. used outdoor experiments to show that future research should account for radio propagation in 3D terrain [59]. To incorporate the effects of terrain when simulating static ad hoc networks, Durkin’s propagation model [60] has been used with digital elevation models (DEMs) or triangulated irregular networks (TINs) to show that terrain can decrease the number of links between nodes [61] and change network connectivity [62]. Simulations using Durkin’s model, DEMs, and mobile nodes have shown that incorporating terrain results in more realistic estimates of network performance [63, 64], that terrain can both degrade and improve performance [63], and that some performance metrics may decrease with increasing moving speed [64]. Loo et al. used a 3D terrain model based on DEMs to estimate path loss and found that the terrain profile between nodes was an important predictor [65]. DEMs have also been used to develop methods that optimize the placement of WSNs for wildfire monitoring [66].

As is evident in prior literature, the majority of past research has emphasized use of simulation modeling methods that require assumptions about network propagation and routing protocols. An alternative approach to studying the connectivity of mesh networks in complex, forested landscapes that has received comparatively little attention is the use of remote sensing to characterize and map connectivity. Al-Turjman et al. mention using lidar to characterize trees and forests in order to determine potential node positions for optimal network deployment [67]. In order to link remote sensing data to measured radio frequency path loss, Jiang et al. used the Normalized Difference Vegetation Index (NDVI) derived from Landsat 8 satellite imagery to predict the path loss exponent [68]. Demetri et al. expanded on the model presented by Azevedo and Santos [57] to propose a method for using lidar to predict signal attenuation, and validated predictions using WSN deployments in forests [69]. This model was subsequently used to develop an automated approach to identifying optimal node placement in forested environments [70]. Oroza et al. developed a machine learning program that uses lidar data to identify potential locations for snow sensors [71]. Additionally, received signal strength indicator (RSSI) measurements from the American River Hydrologic Observatory were used to train a path loss model using machine learning [72]. A variety of independent variables were used in the model, including path ground distance, canopy coverage, terrain complexity, and path angle, many of which were extracted from DEMs or the National Land Cover Database [72]. Prediction error was lower using machine learning when compared to traditional empirical path loss model approaches [72].

The effects of terrain and vegetation on mesh network connectivity have been evaluated independently, but the potential interactions and relative importance of the two have not been quantified. In this study, we developed a method to predict mesh network connectivity using remote sensing data without relying on traditional network simulators. We performed a large field experiment to test the connectivity of a network of goTenna Pro devices paired with Google Pixel smartphones. Dirichlet regression was used to predict connectivity using terrain and vegetation metrics as predictors. The metrics were derived from lidar data, satellite imagery, and a combination of the two. Our first research objective was to characterize the connectivity of mesh networks in variable forest and topographic conditions in the northern Rocky Mountain region of the United States. Our second objective was to evaluate the abilities of lidar and satellite remote sensing data to predict connectivity, as evident in useful predictive regression relationships. We hypothesized that using both lidar- and satellite-derived metrics would improve model predictions. Our third objective was to assess the relative importance of the different predictive metrics. In doing so, we hypothesized that terrain-related metrics would be more important than vegetation metrics for predicting connectivity, as indicated by how frequently these two types of variables were selected for inclusion in final models. This work will inform use of goTenna Pro and other ad hoc mesh radio networks for resource monitoring in wildland firefighting, forestry, and public safety, and will allow others to predict the connectivity of these networks using publicly available remote sensing data.

## Materials and methods

### Ethics statement

Field data was collected on a mixture of public and private land. All landowners were contacted prior to sampling. Landowners were provided a general description of study methods and verbal permission to access their land was obtained.

### Field study

**Fig 1:**
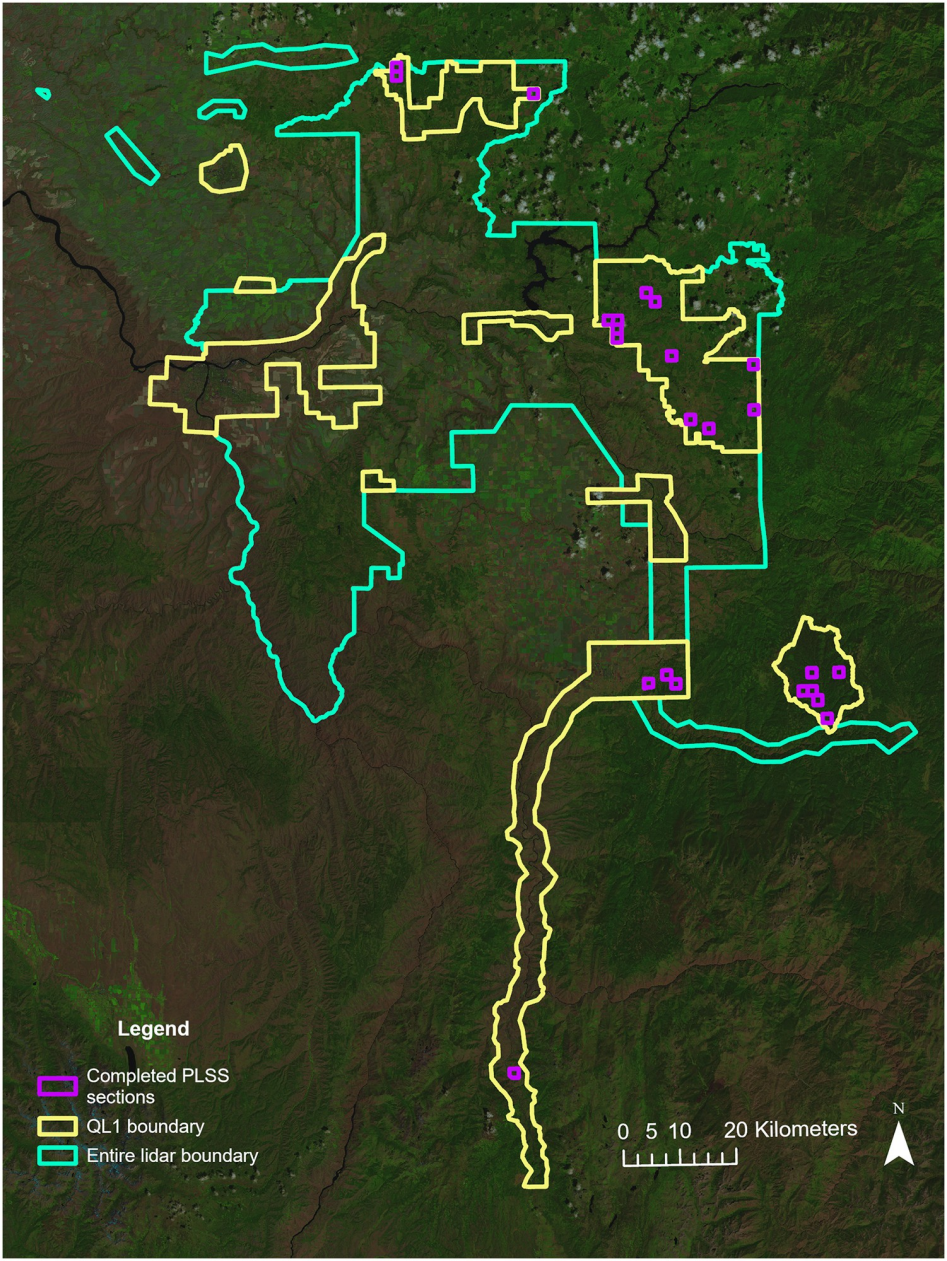
Map of the area covered by the entire *Clearwater–Nez Perce 3DEP 2016* lidar acquisition, highlighting the QL1 data boundaries and the 24 randomly-selected PLSS sections. Background map is a composite of Landsat 8 Operational Land Imager (OLI) Collection 2 Tier 1 Level-2 Science Product (L2SP) surface reflectance scenes (bands 4, 5, and 6) downloaded from USGS EarthExplorer [77]. Fig 1 was created using ArcGIS Pro version 3.0 from Esri.

In order to evaluate the terrain and vegetation factors affecting the connectivity of VHF-based mesh networks, a designed field experiment was conducted on 24 sections delineated by the Public Land Survey System (PLSS). Sections are approximately one square mile in size and were selected from within the boundary of the *Clearwater–Nez Perce 3DEP 2016* lidar acquisition covering 2,662 square miles in northwestern Idaho and southeastern Washington [73]. In an effort to categorize airborne lidar datasets, the National Enhanced Elevation Assessment (NEEA) defined five elevation data Quality Levels (QLs) characterized by horizontal resolution and vertical accuracy [74]. The United States Geological Survey (USGS) National Geospatial Program (NGP) established the 3D Elevation Program (3DEP) based on the NEEA recommendations [75]. Of the five quality levels, Quality Level 1 (QL1) and Quality Level 2 (QL2) are considered acceptable for 3DEP and the standard national DEM available through The National Map [75]. QL1 data has an aggregate nominal pulse spacing of ≤ 0.35 m and aggregate nominal pulse density of ≥ 8.0 pls/m^2^ [75]. QL2 data has an aggregate nominal pulse spacing of ≤ 0.71 m and aggregate nominal pulse density of ≥ 2.0 pls/m^2^ [75]. Both QL1 and QL2 have vertical accuracies (RMSE_z_) of ≤ 10 cm [75]. Both QL1 and QL2 lidar was flown for the *Clearwater–Nez Perce 3DEP 2016* acquisition between October 29, 2016, and November 13, 2016, with the QL1 data covering 847 square miles and the QL2 data covering 1,815 square miles. This study was confined to the area covered only by QL1 data (Fig 1). The QL1 data was flown at an altitude of 1,900 m with a 60° field of view. The resulting area had an average point density of 9.5 pts/m^2^ and average point spacing of 0.35 m. Quantum Spatial (St. Petersburg, FL, USA) processed the QL1 data into a 0.5-m hydro-flattened bare earth raster DEM. All lidar data was downloaded using the Globus Web App [76].

In order to capture variability in terrain, sections were selected based on the rumple index, which can be used to characterize ground roughness [78]. The 0.5-m DEMs were used to calculate the rumple index using the rumple_index function from the lidR package (version 3.2.3) [79] in the R statistical programming environment (version 4.1.2) [80]. This function calculates the ratio between a surface’s area and its projected area on the ground. A rumple index was calculated for each section covered completely by the QL1-derived 0.5-m DEMs. Rumple indices for sections covered by the entire QL1 dataset ranged from 1.005996 to 1.291691, although sections with rumple indices > 1.19 were excluded due to limited accessibility. Sections were visually inspected in Google Earth, and sections that 1) appeared to be less than 25% forested; 2) had major river crossings; or 3) included any urban areas were removed from consideration. While PLSS sections are supposed to be one square mile (260 hectares [ha]) in area, the actual areas vary and only sections that were within 10% (26 ha) of this were included. After filtering sections by these criteria, the number of eligible sections was reduced from 626 to 286, with rumple indices ranging from 1.005997 to 1.188209. This reduced range was divided into five categories (1.005997–1.042439; 1.042439–1.078882; 1.078882–1.115324; 1.115324–1.151767; 1.151767–1.188209) and five sections from each category were randomly selected (Fig 1). Sections were located on a mix of public and private land, and only sections where landowner permission was granted were utilized. Only seven of the 286 eligible sections fell into the highest rumple category (1.151767–1.188209). Two of these were located on private land, and landowner permission was not granted. A third section was inaccessible due to hunting restrictions, weather, and its remote location. This resulted in only four eligible sections in the highest rumple category and meant a total of 24 sections were used in the study.

Field data was collected between June 14, 2019, and November 1, 2019. Within each section, five goTenna Pro units paired with Google Pixel smartphones were placed in randomly-selected locations. In order to ensure goTennas were not too close together, the distance between all goTenna locations within each section was calculated prior to sampling and new locations were randomly selected whenever the distance between devices was less than 75 m. goTenna Pros operate on tunable VHF frequencies between 142 and 175 MHz, with options to configure both control and data channels. For this study, the Pros were set up with two control channels (151.7000 MHz and 151.7600 MHZ) and one data channel (151.5125 MHZ) licensed to our lab group. Power output was set to 5 W and the bandwidth was 11.8 kHz. When paired with a smartphone, users can send text messages and share locations (acquired from the smartphone’s GNSS chip) through the goTenna. All Pro/Pixel pairs were mounted 1 m above the ground on wooden stakes and were set to transmit their coordinates to each other every 30 seconds. Data was recorded using the Android Team Awareness Kit–Civil Use (ATAK-CIV) app, version 3.8.1 [81] and the goTenna ATAK-CIV Plugin. Because of the way in which ATAK-CIV records location data, it is impossible to detect missed locations unless you know the tracked unit is moving and expect a change in coordinates every 30 seconds. Thus, a sixth goTenna Pro paired with a Pixel was carried on a belt by a volunteer who traversed each section once diagonally. The path began at a randomly-selected corner of the section and the volunteer walked for at least one hour. Due to the variable nature of the terrain and vegetation, the time it took to traverse each section varied. As a result, the number of total transmissions in each section ranged from 129 to 327. The true coordinates of this mobile unit were considered to be the coordinates recorded locally and were used to determine whether each new location was successfully transmitted to the five stationary Pros. Thus, for each section we calculated the proportion of the total transmitted locations that were received by each of the stationary Pros. Additionally, there were occasional instances when the mobile goTenna did not record a change in coordinates for 30 seconds or longer, such as when GNSS signal quality was poor or when the volunteer stopped moving for a brief period. This would then be detected as an incorrect missed signal when looking at the data recorded by the stationary units. To avoid this, the dataset was visually inspected to ensure that all missed signals were due to missed transmissions, and not due to a lack of change in coordinates from the mobile unit. Finally, one of the stationary goTenna Pros turned off for an unknown reason during data collection in one PLSS section. This occurred when the volunteer carrying the mobile goTenna was more than halfway through traversing the section and as a result, connectivity data was calculated only up until the point at which the stationary goTenna turned off.

### Remote sensing data processing

#### Lidar metrics

**Fig 2:**
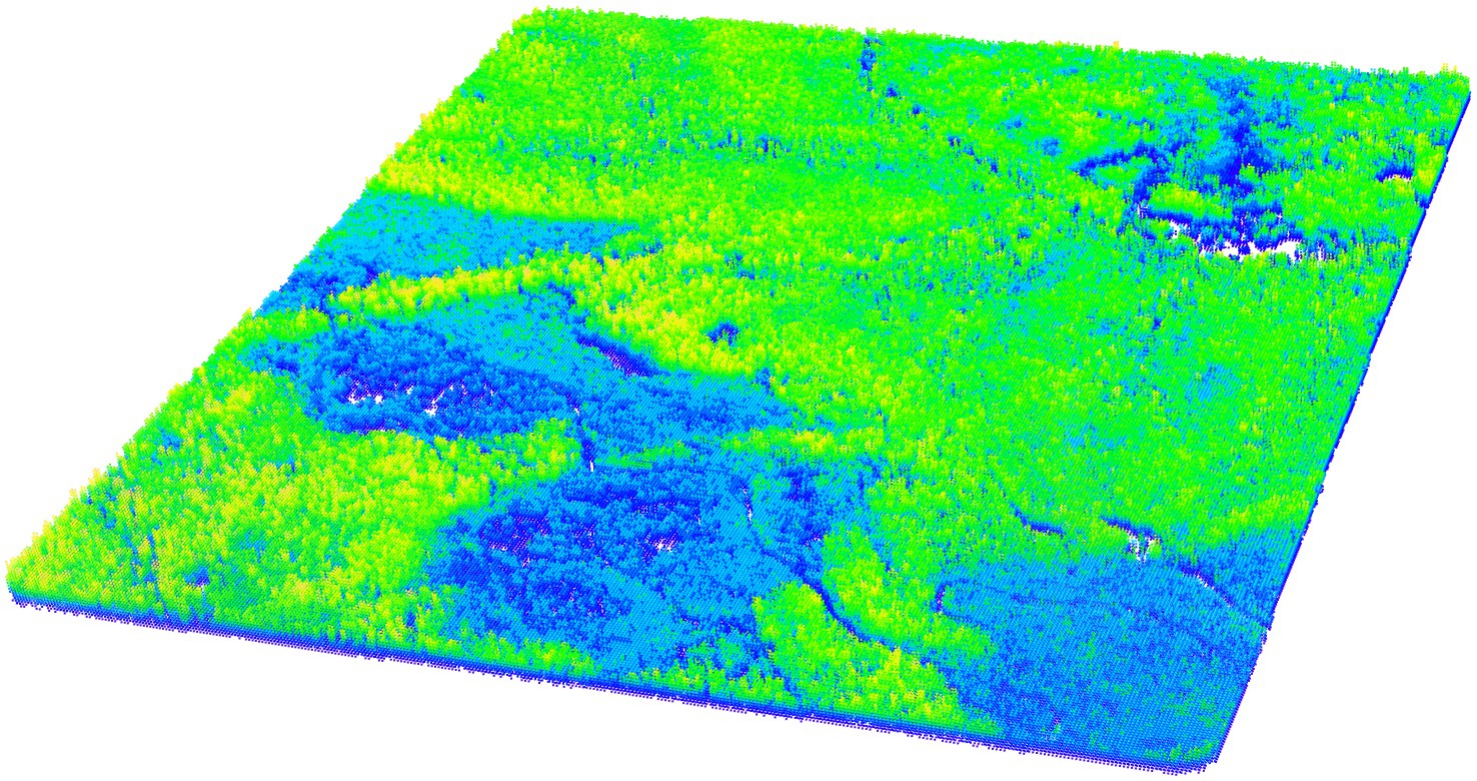
Example PLSS section illustrating an intermediate step in the voxel-based metric calculation process. The section has been divided horizontally and vertically into 5-m resolution voxels. Statistics were then calculated to summarize points contained within each voxel. In this example, each point represents the mean height within the respective 5-m voxel. This data is used to subsequently calculate the voxel-based metrics shown in Table 1 for the entire PLSS section.

In order to predict connectivity, a variety of metrics were derived from the QL1 lidar data as well as from Landsat 8 satellite imagery in order to represent the potential effects of terrain, vegetation, and canopy cover. All lidar data was processed in R, version 4.1.2 [80] and a variety of metrics based on the DEMs, canopy height models (CHMs), point cloud, and voxels were calculated. To characterize terrain roughness, the 0.5-m DEMs were used to calculate the rumple index for each PLSS section as described above (Table 1). The DEMs were also used to calculate the surface relief ratio (SRR) as well as the mean and standard deviation of slope, topographic position index (TPI), roughness, flow direction, hierarchical slope position (HSP), McNab’s curvature, terrain ruggedness index (TRI), heat load index (HLI), and dissection (Table 1). Vegetation height was calculated by normalizing the z-values of the lidar point cloud using the normalize_height function in the R lidR package (version 3.2.3) [79] together with the 0.5-m DEMs. The normalized point cloud was cropped for each PLSS section and subsequently used to calculate a variety of metrics to characterize vegetation and canopy cover. First, the point cloud was visualized for each section and any remaining obvious outliers were removed manually. The normalized point cloud was then used to create a 0.5 m pit-free CHM using the lidR grid_canopy function, using only first vegetation returns ≥ 0.27 m in height. The lidR rumple_index function was then used with the resulting CHM to calculate a rumple index for the canopy, in order to characterize the irregularity and topography of the canopy surface [78, 82]. The percentage of returns classified as “ground” was also calculated using the lidR cloud_metrics function using all vegetation returns ≥ 0.27 m and the lidR LAD function was used to calculate leaf area density using 1 m height bins from 2.5 m to 30.5 m using all, first, and last vegetation returns ≥ 0.27 m (Table 1). Finally, custom functions [83] developed by Blackburn et al. [84] were used to calculate a variety of cloud- and voxel-based metrics (Table 1). Both the point cloud, or area-based, and voxel-based variables summarized aspects of point density, height, and intensity across each section. Voxels, or volumetric pixels, are created by dividing the lidar point cloud along the horizontal and vertical axes, and voxel-based metrics are then calculated by summarizing the points encompassed by each voxel (Fig 2) [85]. The Blackburn std_cloud function [83] was used within the lidR cloud_metrics function to calculate a variety of cloud-based metrics using all, first, and last vegetation returns ≥ 0.27 m (Table 1). The Blackburn std_voxel and vox_mt functions [83] were modified slightly and used within the lidR cloud_metrics function to calculate a variety of voxel-based metrics at 3 m, 4 m, and 5 m resolutions using all vegetation returns ≥ 0.27 m (Table 1). Specifically, the original skewness and kurtosis calculations within both the std_voxel and vox_mt functions were replaced with the skewness and kurtosis functions in the R moments package (version 0.14) [86] and the height thresholds and height bins used to calculate canopy closure (cc_abovez_res) and mean percentage canopy closure (pcc_res) were modified for each resolution (Table 1). In total, 919 lidar metrics were calculated. These lidar DEM-, point cloud-, and voxel-derived metrics were included because they have been shown to be correlated with forest structural parameters [78, 84, 85, 87–103].

**Table 1:** Lidar DEM-, point cloud-, and voxel-derived metrics. RT: all = all vegetation returns ≥ 0.27 m; first = first vegetation returns ≥ 0.27 m; last = last vegetation returns ≥ 0.27 m.

Variable	Description	Source	Classification
**DEM-based**			
Rumple_index	DEM was used to calculate a rumple index for each section using the rumple_index function in the R lidR package (version 3.2.3) [79]	DEM	Topography
SRR	DEM was used to calculate the surface relief ratio (SRR) for the entirety of each section as: (mean(x)—min(x)) / (max(x)—min(x)), where x represents the DEM elevation values [104]	DEM	Topography
Slope_Mean and Slope_SD	DEM was used to create slope rasters for each section in degrees using eight neighbors using the terrain function in the R terra package (version 1.4–22) [105], then the mean and standard deviation of these rasters were calculated for each section	DEM	Topography
TPI_Mean and TPI_SD	DEM was used to create topographic position index (TPI) rasters for each section using the terrain function in the R terra package [105], then the mean and standard deviation of these rasters were calculated for each section. TPI is the difference between the value of a cell and the mean value of its eight surrounding cells [105–107]	DEM	Topography
TRI_Mean and TRI_SD	DEM was used to create terrain ruggedness index (TRI) rasters for each section using the terrain function in the R terra package [105], then the mean and standard deviation of these rasters were calculated for each section. TRI is the mean of the absolute differences between the value of a cell and the value of its eight surrounding cells [105, 107]	DEM	Topography
Rough_Mean and Rough_SD	DEM was used to create roughness rasters for each section using the terrain function in the R terra package [105], then the mean and standard deviation of these rasters were calculated for each section. Roughness is the difference between the maximum and the minimum value of a cell and its eight surrounding cells [105, 107]	DEM	Topography
Flow_Mean and Flow_SD	DEM was used to create flow direction (of water) rasters for each section using the terrain function in the R terra package [105], then the mean and standard deviation of these rasters were calculated for each section. Flow direction is the direction of the greatest drop in elevation (or the smallest rise if all neighbors are higher) [105]	DEM	Topography
HSP_Mean and HSP_SD	DEM was used to create hierarchical slope position (HSP) rasters for each section using the hsp function in the R spatialEco package (version 1.3–7) [108], then the mean and standard deviation of these rasters were calculated for each section. HSP is the hierarchical scale decomposition of the topographic position index and was calculated using rectangular windows ranging in size from 3 cells to 27 cells in 4-cell increments [108, 109]	DEM	Topography
Curv_Mean and Curv_SD	DEM was used to create McNab’s curvature rasters for each section using the curvature function in the R spatialEco package [108], then the mean and standard deviation of these rasters were calculated for each section. This is a variant of the surface curvature (concavity/convexity) index and is confined to the view of a 3 x 3 window [108, 110]	DEM	Topography
HLI_Mean and HLI_SD	DEM was used to create heat load index (HLI) rasters for each section using the hli function in the R spatialEco package [108], then the mean and standard deviation of these rasters were calculated for each section. This function calculates the McCune and Keon 2002 heat load index which estimates potential annual direct incident radiation [108, 111]	DEM	Topography
Diss_Mean and Diss_SD	DEM was used to create dissection rasters for each section using a window size of three and the dissection function in the R spatialEco package [108], then the mean and standard deviation of these rasters were calculated for each section. This function calculates Martone’s modified dissection [108, 112]	DEM	Topography
**CHM-based**			
rumple_index_chm	Point cloud was used to create a 0.5 m pit-free canopy height model (CHM) using the lidR grid_canopy function, using only first vegetation returns ≥ 0.27 m in height. The lidR rumple_index function was then used with the resulting CHM to calculate a rumple index for the canopy [79]	CHM	Height
**Point cloud-based**			
pground_veg27	Percentage of returns classified as “ground” calculated using the lidR cloud_metrics function using all vegetation returns ≥ 0.27 m [79]	Point cloud	Point density
z_x_RT	lidR LAD function was used to calculate leaf area density using 1 m height bins (x = 2.5, 3.5, 4.5, …, 30.5 m) for a given return type (RT = all, first, last) [79]	Point cloud	Point density
**Point cloud-based, calculated using std_cloud function from Blackburn et al**. [83, 84]			
per_RN_x_RT	Percent of points from a given return number (x = return numbers 1–7) and return type (RT = all, last) [84]	Point cloud	Point density
zstat_RT	Height-based descriptive statistics of the point cloud within a section for a given return type (RT). Statistics (stat) = max, mode, mean, quadratic mean, standard deviation, variance, coefficient of variation, IQR, average absolute deviation, skewness, kurtosis, entropy, L-Moments (2–4), L-moment skewness, and L-moment kurtosis. RT = all, first (except for max), last (except for mode) [84]	Point cloud	Height
qHt_x_RT	Height quantiles (x = 1, 5, 10, 15, 20, 25 …, 95, 99) for a given return type (RT = all, first, last) [84]	Point cloud	Height
decilex_RT	Deciles (x = 2, 3, …, 9) for height distributions within a section for a given return type (RT = all, first, last) [84, 85, 89]	Point cloud	Height
dcumx_RT	Cumulative deciles (x = 1, 2, …, 9) for height distributions within a section for a given return type (RT = all, first, last) [84, 85, 89]	Point cloud	Height
pHtBin_x_RT	Percent of points within height bins (x = ≤ 5, 5–10, 10–15, 15–20, …, > 50) for a given return type (RT = all, first, last) [84, 85]	Point cloud	Point density
pz_1r_RT	Percent of first returns above a given height (z = 2m, 10m, 20m, mean height, mode height) for a given return type (RT = all, last [except for percent above mode height]) [84]	Point cloud	Point density
pz_RT	Percent of all returns above a given height (z = 2m, 10m, 20m, mean height, mode height) for a given return type (RT = all, first, last [except for percent above mode height]) [84]	Point cloud	Point density
istat_RT	Intensity-based descriptive statistics of the point cloud within a section for a given return type (RT). Statistics (stat) = total, min, mean, quadratic mean, standard deviation, variance, coefficient of variation, IQR, average absolute deviation, skewness, kurtosis, and entropy. RT = all, first, last [84]	Point cloud	Intensity
icum_qHt_x_RT	Cumulative intensity returned below quantiles (x = 1, 5, 10, 15, 20, 25 …, 95, 99) for a given return type (RT = all, first, last) [84]	Point cloud	Intensity
LCV_RT	Coefficient of L-variation for a given return type (RT = all, first, last). Calculated as: LCV = second L-moment of heights / first L-moment of heights	Point cloud	Height
**Voxel-based, calculated using std_voxel and vox_mt functions from Blackburn et al**. [83, 84]			
z_ s1_s2_res	Height-based descriptive statistics within a voxel (s1) and summarized at the section level (s2) for a given resolution (res = 3m, 4m, 5m). Statistics (s1 and s2) = median, mean, variance, standard deviation, coefficient of variation, IQR, skewness, and kurtosis [84, 85]	Voxel	Height
i_s1_s2_res	Intensity-based descriptive statistics within a voxel (s1) and summarized at the section level (s2) for a given resolution (res = 3m, 4m, 5m). Statistics (s1 and s2) = median, mean, variance, standard deviation, coefficient of variation, IQR, skewness, and kurtosis [84, 85]	Voxel	Intensity
P_Di_s2_res	Section-level descriptive statistics (s2) of the number of returns below each voxel for a given resolution (res = 3m, 4m, 5m). Statistics (s2) = median, mean, variance, standard deviation, coefficient of variation, IQR, skewness, and kurtosis [84, 85]	Voxel	Point density
npoints_above_s2_res	Section-level descriptive statistics (s2) of the number of returns above each voxel for a given resolution (res = 3m, 4m, 5m). Statistics (s2) = mean, variance, standard deviation, coefficient of variation, IQR, skewness, and kurtosis [84, 100].	Voxel	Point density
FR_Di_s2_res	Section-level descriptive statistics (s2) for the frequency ratio of the number of returns above a voxel to the total returns for a given resolution (res = 3m, 4m, 5m). Statistics (s2) = mean, variance, standard deviation, and IQR [84, 85, 100]	Voxel	Point density
pct_fill_vox_res	Percent of voxels with at least one point in the section for a given resolution (res = 3m, 4m, 5m) [84]	Voxel	Point density
ENL_HN_res	Effective number of layers (ENL) measured through different Hill-Numbers (HN = 0D, 1D, 2D) for a given resolution (res = 3m, 4m, 5m) to quantify vertical structure [84, 85, 99]. Computing ENL consists of classifying voxels as empty/not empty and then calculating various diversity indices based on the proportion of filled voxels in each layer in relation to all filled voxels [85, 99]	Voxel	Point density
cc_abovez_res	Canopy closure at different heights based on the percentage of empty voxels above different heights for a given resolution (res = 3m, 4m, 5m) [84, 85, 98]. Height thresholds for 3-m resolution (z) = 3, 6, 9,…, 24 m. Height thresholds for 4-m resolution (z) = 4, 8, 12,…, 24 m. Height thresholds for 5-m resolution (z) = 5, 10, 15, 20, 25 m.	Voxel	Point density
pcc_res	Mean percentage canopy closure based on the ratio of height bin point density to the overall point density within a section for a given resolution (res = 3m, 4m, 5m) [84, 85, 113]. Height bins for 3-m resolution = 1, 3, 6, 9, …, 21 m. Height bins for 4-m resolution = 1, 4, 8, 12, 16, 20 m. Height bins for 5-m resolution = 1, 5, 10, 15, 20 m	Voxel	Point density

#### Landsat 8 satellite metrics

Landsat 8 Operational Land Imager (OLI) Collection 2 Tier 1 Level-2 Science Product (L2SP) scenes covering the 24 sections were downloaded from USGS EarthExplorer [77] using an acquisition date range from June 1, 2019 to November 1, 2019. Only scenes with cloud cover ranging from 0% to 20% were considered eligible. Landsat data was processed in R. Eligible scenes for each section were cropped using the PLSS section boundary, and these resulting images were then used to ensure that less than 5% of the imagery for each section was “not clear” (using the Pixel QA bands) and that less than 5% of the section was classified as high or medium aerosols (using the Aerosol QA bands). After selecting for these criteria, the scene acquired closest in time to the sampling date for each PLSS section was ultimately chosen to be used to calculate a variety of metrics for each section. L2SP surface reflectance scenes for bands 1–7 were rescaled using multiplicative and additive band-specific scale factors [114] in order to calculate percentage reflectance values. These scenes were then masked using the Pixel QA and Aerosol QA bands to remove pixels that were not clear or were classified as high or medium aerosols. Basic surface reflectance metrics (mean and standard deviation) for each section were calculated for bands 1–7 of the resulting masked scenes (Table 2). Mean and standard deviation of NDVI were also calculated for each section using the masked scenes (Table 2). NDVI was calculated using Eq (1):

$$NDVI=\frac{\left(NIR-Red\right)}{\left(NIR+Red\right)}$$ (1)

Where *Red* is the red band (Landsat 8 OLI band 4) reflectance and *NIR* is the near-infrared band (Landsat 8 OLI band 5) reflectance.

**Table 2:** Satellite-derived metrics.

Variable	Description
SR_stat_B	Surface reflectance statistics (stat = mean and standard deviation) for each band (B = 1–7), calculated for each section
NDVI_stat	NDVI statistics (stat = mean and standard deviation) calculated for each section
GLCM_stat_B_win	Eight GLCM texture metrics (GLCM = mean, variance, homogeneity, contrast, dissimilarity, entropy, second moment, and correlation) were calculated for each section using four window sizes (win = 3, 5, 7, 9) for each band (B = 1–7), then two statistics (stat = mean and standard deviation) were calculated for each metric

Finally, grey-level co-occurrence matrix (GLCM) texture metrics were calculated for each section using the glcm function in the R glcm package (version 1.6.5) [115] and the masked Landsat scenes. Specifically, the mean and standard deviation of eight texture measures (mean, variance, homogeneity, contrast, dissimilarity, entropy, second moment, and correlation) were calculated for bands 1–7 using four window sizes (3, 5, 7, and 9) and 64 grey levels (Table 2). A total of 464 satellite metrics were calculated. These satellite-derived metrics were included because they have been shown to be correlated with forest structural parameters [91, 97, 102, 116–123].

### Model development

#### Connectivity

**Fig 3:**
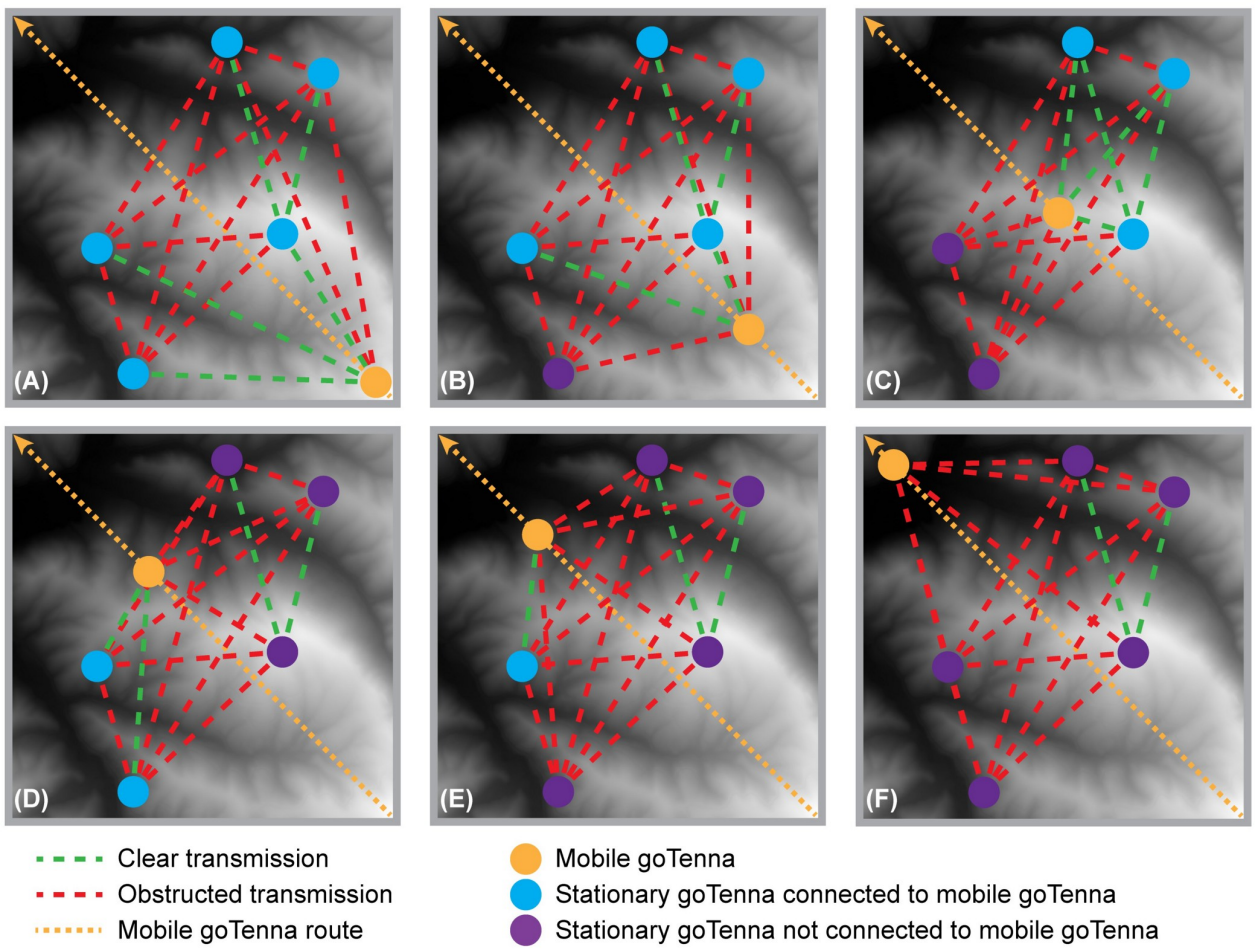
Example locations of the one mobile and five stationary goTenna Pros that may result in each of the six connectivity levels. The mobile goTenna is shown at various locations along the diagonal walking path within a PLSS section. Panels A–F represent connectivity levels Con_6–Con_1, respectively. These connectivity levels correspond to instances in which five, four, three, two, one, or zero stationary devices are connected to the mobile goTenna Pro, respectively.

Connectivity was calculated as the proportion of the total number of transmitted signals sent from the mobile goTenna that were received by five, four, three, two, one, or zero stationary goTennas (Table 3, Fig 3). Specifically, the proportion of total signals received by all five stationary goTennas (Con_6) represented the proportion of time the full network of six devices (i.e., the five stationary goTennas and one mobile goTenna) was connected. The proportion of total signals received by zero stationary goTennas (Con_1) represented the proportion of time none of the stationary goTennas were connected to the mobile goTenna. This resulted in a compositional dataset, where the dependent variables for each section were the proportions of the six connectivity levels, which summed to one. A common problem in compositional data analysis occurs when the dataset contains zero values, since both traditional log-ratio analysis and Dirichlet regression cannot handle zero values in any of the compositional responses. Zeros can be considered to be rounded, essential, or count zeros [124, 125]. Rounded zeros can occur when a component is present but below a detection limit, and it is often suitable to replace rounded zeros by a small value [124]. Essential zeros represent the true absence of that portion of the composition, and it is generally not appropriate to replace these zeros [124]. Instead, various approaches such as combining categories, have been suggested [124]. Count zeros are considered to represent values that may have been observed if a different sampling design or larger number of trials had been utilized [125]. Zero values in our dataset were considered to be count zeros and were replaced using the cmultRepl function in the R zCompositions package (version 1.3.4) [126], which imputes zeros in compositional count datasets based on Bayesian-multiplicative replacement [125, 126]. Specifically, this method imputes zero counts and then multiplicatively adjusts the remaining non-zero components to produce a set of proportions that still sum to one [125, 126].

**Table 3:** Descriptions of the six compositional response variables representing the six connectivity levels and corresponding calculation methods.

Variable	Description	Calculation
Con_6	Proportion of time the full network of six devices (i.e., all five stationary goTennas and one mobile goTenna) were connected	# of signals received by all five stationary goTennas / # of total transmitted signals sent from mobile goTenna
Con_5	Proportion of time five devices (i.e., four stationary goTennas and one mobile goTenna) were connected	# of signals received by four stationary goTennas / # of total transmitted signals sent from mobile goTenna
Con_4	Proportion of time four devices (i.e., three stationary goTennas and one mobile goTenna) were connected	# of signals received by three stationary goTennas / # of total transmitted signals sent from mobile goTenna
Con_3	Proportion of time three devices (i.e., two stationary goTennas and one mobile goTenna) were connected	# of signals received by two stationary goTennas / # of total transmitted signals sent from mobile goTenna
Con_2	Proportion of time two devices (i.e., one stationary goTenna and one mobile goTenna) were connected	# of signals received by one stationary goTenna / # of total transmitted signals sent from mobile goTenna
Con_1	Proportion of time zero stationary goTennas were connected to the mobile goTenna	# of signals received by zero stationary goTennas / # of total transmitted signals sent from mobile goTenna

#### Dirichlet regression

Dirichlet regression is a multivariate generalization of beta regression and can be used to analyze compositional data [127]. It has been used in forestry to model compositional data, specifically to predict forestry planned end products [128], species proportions for forest inventories [129], and biomass component proportions [130–133]. In the common parameterization of the Dirichlet distribution, there is a shape parameter *α*_*c*_ for each of the *c* components. The expected value of any given component *y*_*c*_ is *E*[*y*_*c*_] = *α*_*c*_/*α*_0_, where *α*_*0*_ is the sum of all *α*_*c*_’s. The *α*_*c*_’s are modeled using explanatory variables with a log link using Eq (2):

$$\text{log}(\alpha_{ij})=a_{i}+\beta_{i}z_{j}$$ (2)

Where *z*_*j*_ are the explanatory variables for the *j*th observation (*j* = 1, …, *n*), *a*_*i*_ are the intercepts for the *i*th component (*i* = 1, … *c*), and *β*_*i*_ are the regression coefficients for the *i*th component (*i* = 1, … *c*). The expected values of *α*_*c*_ are then derived as ${\hat{\alpha }}_{ij}=\text{exp}\left({\hat{a}}_{i}+{\hat{\beta }}_{i}z_{j}\right)$. The DirichReg function in the R DirichletReg package (version 0.7–1) [134, 135] was used to fit Dirichlet regression models to the lidar- and satellite-derived terrain and vegetation metrics in order to predict connectivity. Models were fitted using the common parameterization and were created using 1) both lidar- and satellite-derived metrics (LIDSAT); 2) lidar-derived metrics only (LID); and 3) satellite-derived metrics only (SAT). All lidar- and satellite-derived predictor variables were normalized using the maximum and minimum values of each variable. Because so many predictor variables were calculated using the remote sensing data, the Boruta algorithm implemented in the R Boruta package (version 7.0.0) [136] was used to select candidate predictors for each normalized variable set (LIDSAT, LID, SAT). The Boruta algorithm is a feature selection method based on the random forest algorithm [136] and has been used effectively with high dimensional remote sensing data [84, 137]. The Boruta algorithm was run 100 times for each of the six proportion responses (i.e., connectivity levels) and for each of the three variable sets using a maximum number of 1,000 runs each time. The number of times each variable was selected by the algorithm was summed across the 100 iterations. After Boruta feature selection, the number of predictor variables for the six connectivity levels ranged from 17 to 75 for the LIDSAT dataset, 2 to 69 for the LID dataset, and 5 to 27 for the SAT dataset. Because many of these Boruta-selected variables were highly correlated, variables that had correlations > |0.7| were removed and all remaining variables were used to build the initial models. Because each component is allowed to have different explanatory variables when fitting Dirichlet regression models under the common parameterization, all initial models were built using the uncorrelated Boruta-selected variables specific to each connectivity level. As a result, the initial model built with the LIDSAT dataset had between 3 to 12 predictors for each proportion response, the initial model built with the LID dataset had between 1 to 10 predictors for each proportion response, and the initial model built with the SAT dataset had between 2 to 5 predictors for each proportion response (Table 4). For each of the three initial models, variables were removed one at a time in order of highest *p*-values until arriving at the null model with only intercepts for each proportion level. All models created for each dataset were arranged in order of increasing number of parameters and were compared using analysis of variance (ANOVA), which when used in the DirichletReg package implements a likelihood ratio test to perform pairwise tests of Dirichlet regression models. Models were iteratively removed through ANOVA until a final model for each dataset was selected. These three final models were evaluated by four statistics using leave-one-out cross validation (LOOCV): mean absolute error (MAE), root mean squared error (RMSE), mean bias, and mean relative bias (bias%) using Eqs (3–6).

$$MAE=\frac{\sum_{i=1}^{n}\left|Y_{i}-{\hat{Y}}_{i}\right|}{n}$$ (3)

$$RMSE=\sqrt{\frac{\sum_{i=1}^{n}{\left(Y_{i}-{\hat{Y}}_{i}\right)}^{2}}{n}}$$ (4)

$$bias=\frac{\sum_{i=1}^{n}\left(Y_{i}-{\hat{Y}}_{i}\right)}{n}$$ (5)

$$bias\text{\%}=\frac{bias}{\bar{Y}}$$ (6)

Where *n* is the number of observations, *Y*_*i*_ is the *i*th observed proportion, ${\hat{Y}}_{i}$ is the *i*th predicted proportion from the model fitted using the (*n*–1) data, and $\bar{Y}$ is the mean of the observed proportions.

**Table 4:** Uncorrelated Boruta-selected variables specific to each connectivity level used to build initial LIDSAT, LID, and SAT Dirichlet regression models.

Model	Connectivity level	Initial predictors^a^
**LIDSAT**	**Con_6**	SR_SD_B6, variance_Mean_B6_5, variance_SD_B5_3, z_IQR_kurt_4m, i_var_kurt_3m, npoints_above_var_3m
	**Con_5**	i_cv_var_3m, dcum7_last, contrast_SD_B5_9, imin_first, icum_qHt_10_first, FR_Di_mean_5m, per_RN_7_last, z_kurt_skew_5m
	**Con_4**	correlation_Mean_B5_3, i_skew_kurt_4m, npoints_above_mean_4m, i_cv_skew_5m, SR_Mean_B2, icum_qHt_15_last, imin_all, homogeneity_Mean_B5_7
	**Con_3**	z_18.5_last, i_med_skew_3m, npoints_above_IQR_3m, SR_SD_B2, dcum2_all, P_Di_kurt_3m
	**Con_2**	dissimilarity_Mean_B1_9, SR_Mean_B1, SR_SD_B3
	**Con_1**	Slope_Mean, iskew_first, z_skew_mean_3m, i_var_kurt_3m, SR_SD_B2, HSP_Mean, i_skew_IQR_4m, icum_qHt_85_last, mean_Mean_B5_7, P_Di_sd_4m, second_moment_Mean_B4_3, zIQR_last
**LID**	**Con_6**	P_Di_cv_5m, i_sd_kurt_3m, npoints_above_var_5m, SRR, z_IQR_kurt_4m
	**Con_5**	icum_qHt_5_first, decile8_last, i_skew_med_4m, icum_qHt_75_all, i_skew_var_3m, imin_first
	**Con_4**	npoints_above_mean_4m, i_skew_kurt_4m, icum_qHt_15_last, i_cv_skew_5m
	**Con_3**	i_med_skew_3m, z_18.5_last, npoints_above_IQR_3m, P_Di_kurt_4m, z_skew_cv_3m
	**Con_2**	z_cv_kurt_4m
	**Con_1**	Curv_SD, iskew_first, P_Di_var_3m, z_IQR_kurt_4m, HLI_Mean, i_var_kurt_3m, icum_qHt_90_last, npoints_above_kurt_3m, p20_1r_last, z_9.5_last
**SAT**	**Con_6**	variance_Mean_B6_5, correlation_SD_B6_3, variance_SD_B5_3
	**Con_5**	contrast_SD_B5_3, contrast_Mean_B7_7
	**Con_4**	correlation_Mean_B5_7, SR_Mean_B2, second_moment_SD_B5_3
	**Con_3**	SR_Mean_B7, correlation_SD_B3_5, dissimilarity_SD_B3_9, variance_SD_B6_5
	**Con_2**	SR_SD_B5, SR_Mean_B1, second_moment_SD_B7_3, contrast_Mean_B1_3, SR_SD_B6
	**Con_1**	correlation_Mean_B5_9, mean_Mean_B5_3, entropy_SD_B1_3, dissimilarity_Mean_B5_9, variance_SD_B7_5

^a^ Predictor variable names correspond to the lidar-derived metrics listed in Table 1 and to the satellite-derived metrics listed in Table 2.

## Results

### Connectivity summary

Summary statistics of the six connectivity levels showed that the mean proportion of time all five stationary devices were connected to the mobile goTenna (Con_6) was 0.326 (SD = 0.332), the mean proportion of time four of the stationary devices were connected to the mobile goTenna (Con_5) was 0.141 (SD = 0.157), the mean proportion of time three of the stationary devices were connected to the mobile goTenna (Con_4) was 0.106 (SD = 0.133), the mean proportion of time two of the stationary devices were connected to the mobile goTenna (Con_3) was 0.110 (SD = 0.121), the mean proportion of time one of the stationary devices was connected to the mobile goTenna (Con_2) was 0.136 (SD = 0.168), and the mean proportion of time zero stationary devices were connected to the mobile goTenna (Con_1) was 0.182 (SD = 0.125) (Table 5).

**Table 5:** Summary statistics of the six connectivity levels of the dependent variable (proportion of time connected).

Connectivity level	Mean	SD	Range
Con_6	0.326	0.332	0.000–0.905
Con_5	0.141	0.157	0.000–0.506
Con_4	0.106	0.133	0.008–0.500
Con_3	0.110	0.121	0.004–0.406
Con_2	0.136	0.168	0.000–0.559
Con_1	0.182	0.125	0.000–0.445

### Boruta results

**Fig 4:**
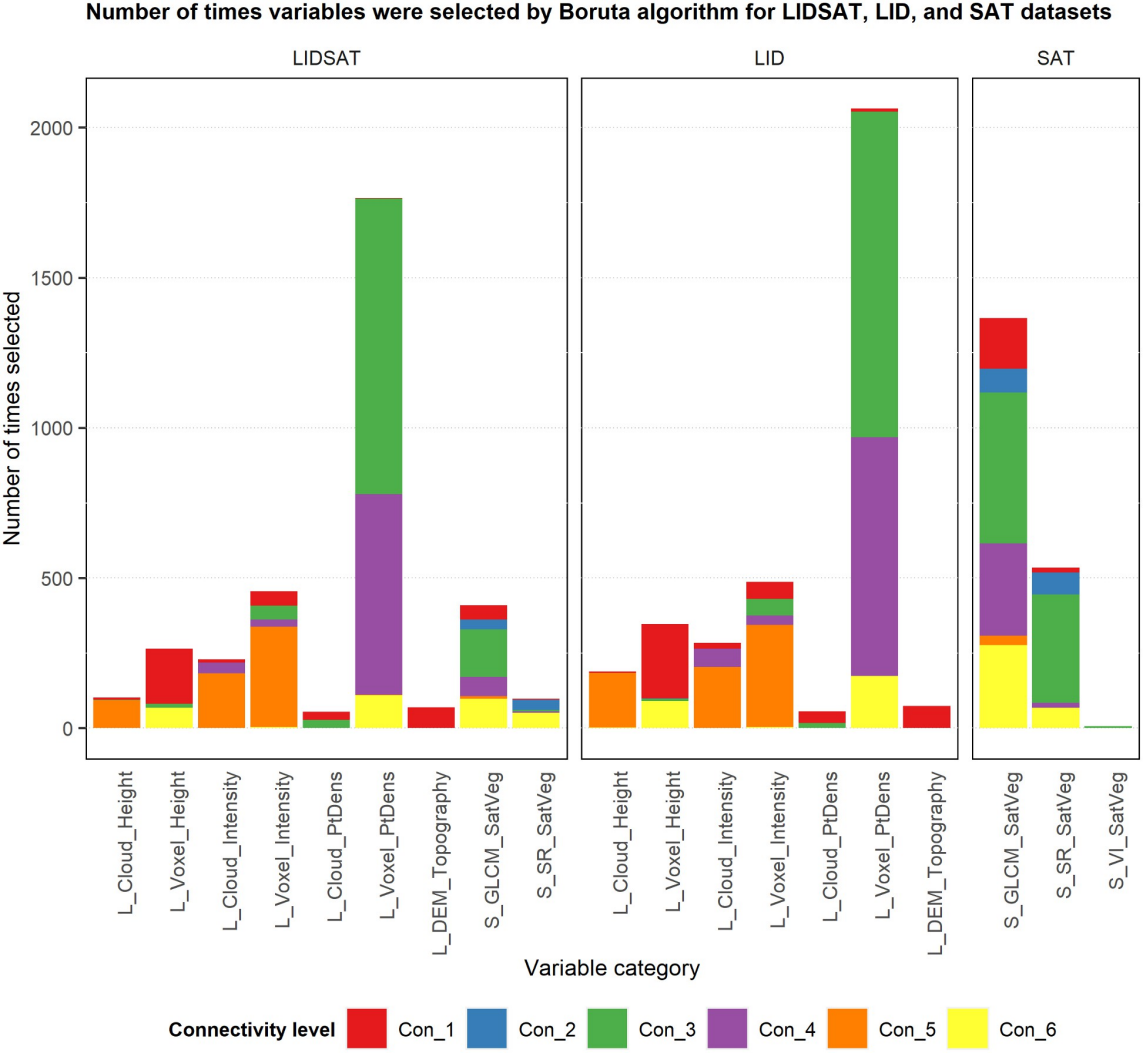
Bar chart of the number of times variables in each category were selected by the 100 iterations of the Boruta algorithm for the LIDSAT, LID, and SAT datasets.

There was high variability in the number of times variables in each category were selected by the Boruta algorithm for each connectivity level over the 100 iterations (Fig 4). For Con_1, lidar voxel height-related metrics were selected most frequently for both the LIDSAT and LID datasets and satellite GLCM texture metrics were selected most frequently for the SAT dataset. For Con_2, only satellite-based GLCM texture and surface reflectance metrics were selected for the LIDSAT and SAT datasets, and only lidar voxel height metrics were selected for the LID dataset. For both Con_3 and Con_4, lidar voxel point density-related metrics were selected most frequently for the LIDSAT and LID datasets. For the SAT dataset, both GLCM texture and surface reflectance metrics were selected relatively frequently for Con_3 and GLCM texture metrics were selected most frequently for Con_4. For Con_5, lidar-derived voxel intensity and cloud intensity metrics were selected most frequently for the LIDSAT dataset, while lidar-derived voxel intensity, cloud intensity, and cloud height metrics were all selected relatively frequently for the LID dataset. Only satellite GLCM texture metrics were selected for the SAT dataset for Con_5, and relatively infrequently. For Con_6, lidar voxel point density and voxel height metrics as well as satellite GLCM texture metrics were selected most frequently for the LIDSAT dataset, while voxel point density and voxel height metrics were selected most frequently for the LID dataset. For the SAT dataset, GLCM texture metrics were selected most frequently for Con_6. Additionally, among the categories, lidar voxel point density metrics were selected much more frequently than variables in other categories. These metrics were selected most frequently for Con_3 and Con_4. Satellite GLCM texture metrics were also selected very frequently and for all six connectivity levels, but most often for Con_3.

**Fig 5:**
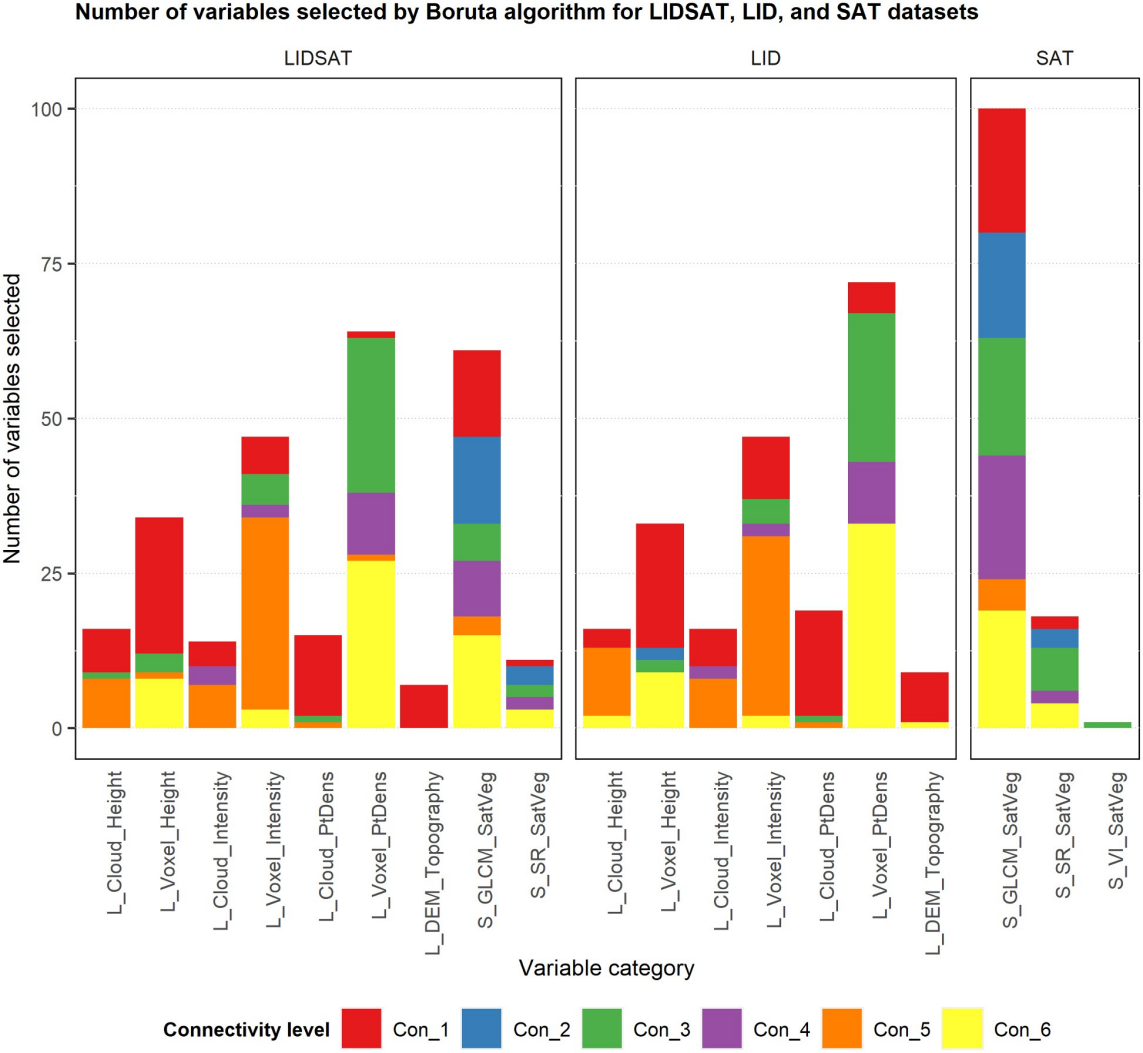
Bar chart of the number of variables from each category that were selected by the 100 iterations of the Boruta algorithm for the LIDSAT, LID, and SAT datasets.

There was also variability in the number of unique variables from each category that were selected by the Boruta algorithm for each connectivity level over the 100 iterations (Fig 5). For Con_1, a large number of lidar voxel height and lidar cloud point density metrics were selected for the LIDSAT and LID datasets. There were also many satellite GLCM texture metrics selected for Con_1 for the LIDSAT and SAT datasets. For Con_2, there were more satellite GLCM texture metrics selected than surface reflectance metrics for both the LIDSAT and SAT datasets, while only a few lidar voxel height metrics were selected for the LID dataset. For Con_3, there were many lidar voxel point density-related metrics selected for both the LIDSAT and LID datasets, and many GLCM texture metrics were selected for the SAT dataset. For Con_4, a large number of voxel point density metrics and satellite GLCM texture metrics were selected for the LIDSAT dataset, and many voxel point density metrics were selected for the LID dataset. For the SAT dataset, a large number of GLCM texture metrics were selected for Con_4. For Con_5, there were many lidar voxel intensity metrics selected for both the LIDSAT and LID datasets and a relatively smaller number of GLCM texture metrics selected for the SAT dataset. For Con_6, there were many lidar voxel point density metrics selected for both the LIDSAT and LID datasets, and quite a few GLCM texture metrics were selected for both the LIDSAT and SAT datasets.

### Summary of final LIDSAT, LID, and SAT models

The final LIDSAT model fitted to the entire dataset had between one and four predictor variables for each proportion (Table 6). Parameters for all predictor variables were significant (*p* ≤ 0.05). Four of these variables were satellite GLCM texture metrics, two were lidar voxel height metrics, four were lidar voxel intensity metrics, three were lidar voxel point density metrics, and one was a lidar point cloud intensity metric. All predictors are related to vegetation, rather than topography.

**Table 6:** Summary of LIDSAT Dirichlet model regression coefficients for each of the six connectivity levels.

Variable	Model term	Estimate	SE	*z*-value	*p*-value
**Con_6**	(Intercept)	-2.118	0.473	-4.481	**7.446E-06**
	variance_Mean_B6_5	1.278	0.431	2.961	**3.062E-03**
	variance_SD_B5_3	2.817	0.534	5.276	**1.320E-07**
	z_IQR_kurt_4m	1.540	0.552	2.788	**5.297E-03**
**Con_5**	(Intercept)	-0.471	0.402	-1.172	2.412E-01
	i_cv_var_3m	3.431	0.609	5.635	**1.753E-08**
	contrast_SD_B5_9	-1.474	0.739	-1.994	**4.617E-02**
**Con_4**	(Intercept)	-2.905	0.858	-3.386	**7.090E-04**
	i_skew_kurt_4m	2.264	0.689	3.287	**1.013E-03**
	npoints_above_mean_4m	2.400	0.959	2.503	**1.232E-02**
	icum_qHt_15_last	2.260	0.902	2.507	**1.218E-02**
**Con_3**	(Intercept)	-0.363	0.239	-1.518	1.291E-01
	npoints_above_IQR_3m	1.325	0.520	2.550	**1.077E-02**
**Con_2**	(Intercept)	-1.474	0.388	-3.801	**1.440E-04**
	dissimilarity_Mean_B1_9	3.466	0.714	4.850	**1.232E-06**
**Con_1**	(Intercept)	1.859	0.554	3.359	**7.821E-04**
	z_skew_mean_3m	-4.285	1.371	-3.126	**1.769E-03**
	i_var_kurt_3m	-2.389	0.965	-2.475	**1.331E-02**
	i_skew_IQR_4m	2.059	0.868	2.371	**1.775E-02**
	P_Di_sd_4m	-1.620	0.717	-2.260	**2.383E-02**

Coefficients in bold are significant (*p* ≤ 0.05).

The final LID model fitted to the entire dataset had three predictor variables for Con_6, one predictor each for Con_5, Con_4, Con_3, and Con_1, and only an intercept for Con_2 (Table 7). The parameters for six of the seven predictor variables were significant (*p* ≤ 0.05), while the parameter for one predictor for Con_6 (SRR) was not (*p* = 0.08). Three of these variables were lidar voxel intensity metrics, two were lidar voxel point density metrics, and two were lidar DEM-based metrics. The five voxel-based metrics are related to vegetation while the two DEM-based metrics (SRR and HLI_Mean) are related to topography.

**Table 7:** Summary of LID Dirichlet model regression coefficients for each of the six connectivity levels.

Variable	Model term	Estimate	SE	*z*-value	*p*-value
**Con_6**	(Intercept)	1.126	0.724	1.556	1.197E-01
	i_sd_kurt_3m	2.848	0.823	3.462	**5.371E-04**
	npoints_above_var_5m	-1.637	0.818	-2.001	**4.544E-02**
	SRR	-1.574	0.904	-1.740	8.179E-02
**Con_5**	(Intercept)	-0.794	0.316	-2.514	**1.194E-02**
	i_skew_var_3m	2.021	0.667	3.030	**2.444E-03**
**Con_4**	(Intercept)	-0.791	0.313	-2.527	**1.150E-02**
	i_skew_kurt_4m	1.330	0.652	2.040	**4.139E-02**
**Con_3**	(Intercept)	-0.527	0.244	-2.161	**3.073E-02**
	npoints_above_IQR_3m	1.219	0.545	2.237	**2.529E-02**
**Con_2**	(Intercept)	-0.272	0.189	-1.438	1.504E-01
**Con_1**	(Intercept)	1.013	0.394	2.568	**1.023E-02**
	HLI_Mean	-2.143	0.935	-2.292	**2.188E-02**

Coefficients in bold are significant (*p* ≤ 0.05).

The final SAT model fitted to the entire dataset had one predictor variable each for Con_6, Con_5, Con_2, and Con_1 and only an intercept for Con_4 and Con_3 (Table 8). Parameters for all four predictors were significant (*p* ≤ 0.05), and all were GLCM texture metrics related to vegetation.

**Table 8:** Summary of SAT Dirichlet model regression coefficients for each of the six connectivity levels.

Variable	Model term	Estimate	SE	*z*-value	*p*-value
**Con_6**	(Intercept)	-1.308	0.339	-3.862	**1.125E-04**
	variance_SD_B5_3	3.201	0.592	5.409	**6.323E-08**
**Con_5**	(Intercept)	0.325	0.301	1.081	2.797E-01
	contrast_SD_B5_3	-1.398	0.656	-2.131	**3.306E-02**
**Con_4**	(Intercept)	-0.442	0.192	-2.304	**2.123E-02**
**Con_3**	(Intercept)	-0.352	0.191	-1.848	6.457E-02
**Con_2**	(Intercept)	-0.977	0.335	-2.919	**3.514E-03**
	contrast_Mean_B1_3	1.857	0.739	2.512	**1.200E-02**
**Con_1**	(Intercept)	0.741	0.347	2.131	**3.305E-02**
	mean_Mean_B5_3	-1.573	0.696	-2.258	**2.396E-02**

Coefficients in bold are significant (*p* ≤ 0.05).

### Evaluation of final LIDSAT, LID, and SAT models

LOOCV showed that MAE ranged from 0.082 to 0.249 for the LIDSAT model, from 0.081 to 0.258 for the LID model, and from 0.102 to 0.256 for the SAT model (Table 9). RMSE ranged from 0.101 to 0.314 for the LIDSAT model, from 0.103 to 0.310 for the LID model, and from 0.121 to 0.313 for the SAT model (Table 9). Bias ranged from -0.048 to 0.073 for the LIDSAT model, from -0.030 to 0.083 for the LID model, and from -0.029 to 0.092 for the SAT model (Table 9). Bias% ranged from -26.2% to 22.5% for the LIDSAT model, from -27.1% to 25.5% for the LID model, and from -19.6% to 28.2% for the SAT model (Table 9). Bias was negative for Con_5, Con_4, Con_3, and Con_1 in all three final models as well as for Con_2 in the SAT model (Table 9), meaning these models tended to overpredict the proportion. Bias was positive for Con_6 in all three final models and for Con_2 in the LIDSAT and LID models (Table 9), meaning these models tended to underpredict the proportion.

**Table 9:** Accuracy metrics calculated for each connectivity level using LOOCV.

		Connectivity level					
Metric	Model	Con_6	Con_5	Con_4	Con_3	Con_2	Con_1
**MAE**	**LIDSAT**	0.249	0.083	0.084	0.082	0.119	0.123
	**LID**	0.258	0.106	0.093	0.081	0.137	0.091
	**SAT**	0.256	0.116	0.107	0.102	0.130	0.104
**RMSE**	**LIDSAT**	0.314	0.117	0.110	0.101	0.157	0.159
	**LID**	0.310	0.133	0.111	0.103	0.173	0.110
	**SAT**	0.313	0.145	0.129	0.122	0.162	0.121
**bias**	**LIDSAT**	0.073	-0.005	-0.019	-0.019	0.017	-0.048
	**LID**	0.083	-0.019	-0.021	-0.030	0.013	-0.026
	**SAT**	0.092	-0.019	-0.015	-0.022	-0.007	-0.029
**bias%**	**LIDSAT**	22.5	-3.7	-17.8	-16.8	12.5	-26.2
	**LID**	25.5	-13.2	-20.3	-27.1	9.5	-14.3
	**SAT**	28.2	-13.7	-14.0	-19.6	-4.8	-16.2

**Fig 6:**
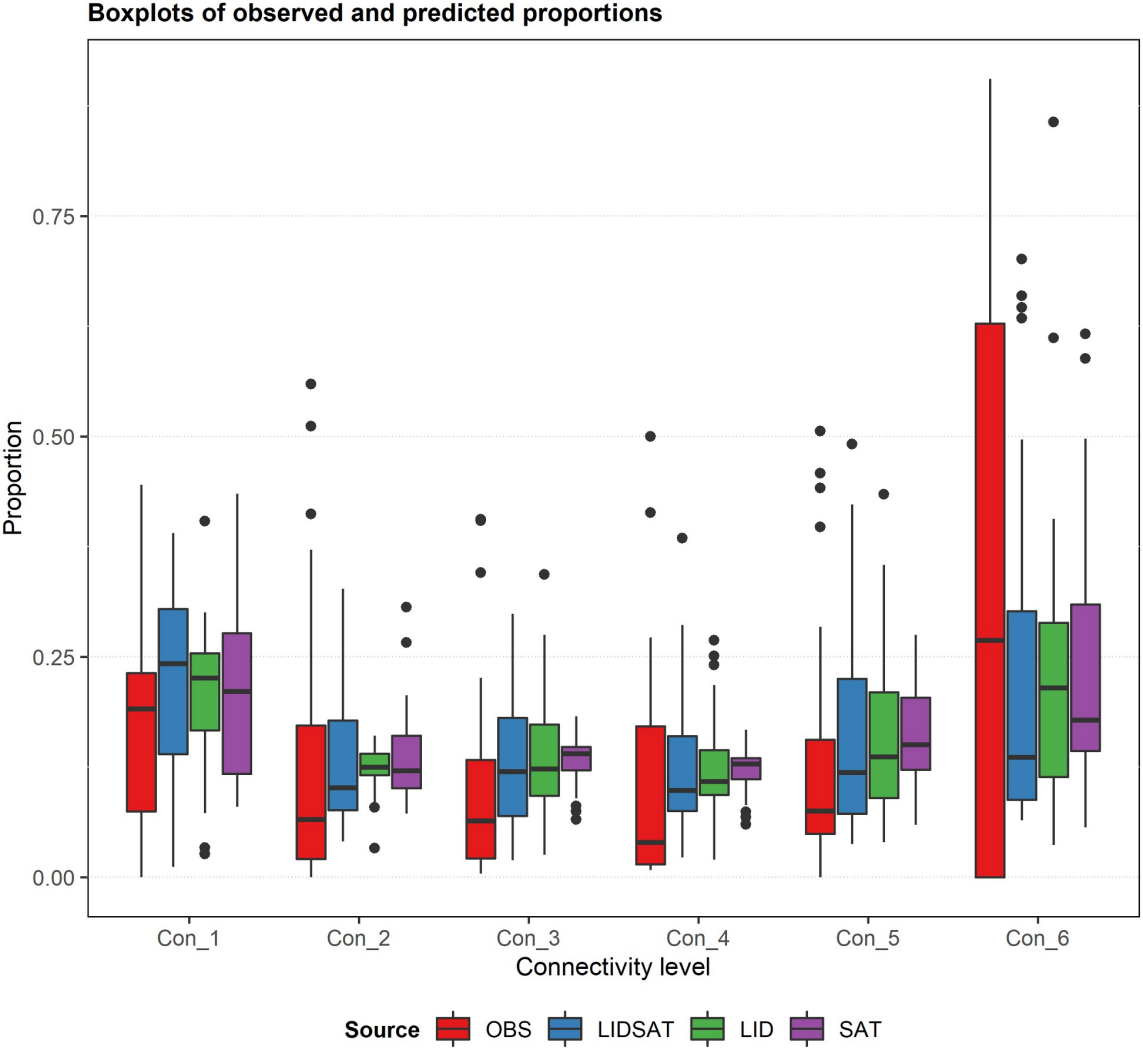
Boxplots of observed and predicted proportions of the six connectivity levels. Predictions were obtained using the final LIDSAT, LID, and SAT models fitted to the entire dataset.

Boxplots of the observed and predicted proportions calculated using the final LIDSAT, LID, and SAT models fitted to the entire dataset illustrate the wide variability in the data, especially for the Con_6 connectivity level (Fig 6). The boxplots further illustrate how the three final models tended to overpredict the Con_5, Con_4, Con_3, and Con_1 connectivity levels (Fig 6). The boxplots also show how all three final models tended to underpredict the Con_6 connectivity level (Fig 6).

## Discussion

In this study, we characterized the connectivity of smartphone-based goTenna mesh radio networks for location sharing in forests using lidar and satellite remote sensing data and developed Dirichlet regression models to predict the connectivity of these networks over a range of vegetation and topographic conditions. Our results showed that for a network of six goTenna Pros composed of one mobile device and five stationary devices deployed at randomized locations within PLSS sections approximately 260 ha in area, the full network was connected, on average, only 32.6% of the time and the mobile goTenna Pro was disconnected from all other devices 18.2% of the time. There was also a wide range in the proportion of time all six devices were connected (0.000–0.905). The network was never fully connected in some sections, while in others, the full network was connected 90.5% of the time. Similarly, the mobile goTenna was not connected to any other device between 0% and 44.5% of the time, depending on the section. This high variability in network connectivity has important implications for wildland fire incident command and other public health and safety applications of mesh networks in forests. Wildland fire managers, search and rescue personnel, forestry and logging workers, and others relying on these networks for improved SA and safety should anticipate gaps in connectivity during which one or more individuals may not be able to communicate or share locations on the network.

The variables most commonly chosen by the Boruta variable selection algorithm were lidar voxel point density metrics as well as voxel intensity metrics for the two datasets containing lidar data (LIDSAT and LID). The lidar DEM-based topography variables were selected much less frequently than the lidar point cloud and voxel predictors more related to vegetation. Satellite GLCM metrics were selected more frequently by the Boruta algorithm compared to the surface reflectance and NDVI metrics for the two datasets containing satellite data (LIDSAT and SAT). The majority of predictors in the final LIDSAT model were lidar voxel metrics, which represented aspects of lidar intensity, point density, and canopy height. However, a variety of satellite GLCM texture variables were also present in the final LIDSAT model, suggesting that both lidar and satellite data were important or complementary predictors of connectivity. Most of the predictors in the final LID model were lidar voxel metrics representing aspects of intensity and point density. However, this model also had two lidar DEM variables related to topography, which is in contrast to the LIDSAT model that depended primarily on vegetation. The final SAT model only had GLCM texture variables, suggesting that these were better predictors of connectivity than surface reflectance or NDVI. While we had anticipated that topography would be an important predictor of connectivity, the relatively minor presence of DEM predictors in both the Boruta variable selection results and the final models shows that vegetation more directly affected network connectivity. This could in part be due to the relationship between the size of each PLSS section and the number of devices deployed. For example, it is possible that using a network of six devices in an approximately 260-ha area was enough to overcome some of the effects of topography on radio signal attenuation, and that vegetation, which is known to affect near-ground peer-to-peer radio signal propagation [46, 50, 52–54], most impacts connectivity. Future work should evaluate the relationship between connectivity, number of devices, and study area.

Both the final LID and SAT models had connectivity levels without a predictor variable, which suggests that some connectivity levels may have been related only to the lidar or satellite data. In particular, Con_2 was modeled with only an intercept in the final LID model but had a significant satellite predictor in both the LIDSAT and SAT models. This suggests that this level of connectivity (i.e., the proportion of time that the mobile goTenna was connected to just one stationary device) could be modeled with satellite data better than lidar data. Both Con_3 and Con_4 only had intercepts in the SAT model but had significant lidar-based predictors in both the LIDSAT and LID models, indicating that these two levels of connectivity (i.e., the proportion of time the mobile goTenna was connected to either two or three stationary devices) could be modeled with lidar data better than satellite data. This is somewhat surprising based on the Boruta results, in which satellite GLCM and surface reflectance metrics were selected commonly for Con_3 and satellite GLCM metrics were selected commonly for Con_4. In contrast to the LID and SAT models, the LIDSAT model had significant predictors for each connectivity level, suggesting that using both data sources may be more useful than relying on just lidar or satellite data alone for prediction.

MAE, RMSE, and bias were all worse for the Con_6 connectivity level compared to the other connectivity levels for all three final models. Bias% was worse for the Con_1, Con_3, and Con_6 connectivity levels for the LIDSAT, LID, and SAT models, respectively, compared to the other connectivity levels. These accuracy metrics were slightly better for the LIDSAT model compared to the other two models for most proportion levels, but worse than the other two models for the Con_1 connectivity level. This suggests that both the LID and SAT models predicted the proportion of time that the mobile goTenna Pro was not connected to any other device somewhat better than the model using both data sources, but that the LIDSAT model predicted the proportion of time that the mobile device was connected to one or more stationary devices slightly better than either the LID or SAT model in most cases. Finally, all three models tended to underpredict the proportion of time that the mobile goTenna Pro was connected to all five stationary devices (Con_6) and to overpredict the proportion of time the mobile goTenna Pro was not connected to any other device (Con_1). This suggests that utilizing these models in practice may lead to underestimating the proportion of time that the full network of devices is expected to be connected and overestimating the proportion of time that the mobile goTenna is expected to be disconnected from all other devices.

In practice, wildland fire incident command teams may place a device on a high point such as an overhead aircraft to serve as a relay node and potentially improve connectivity. The effect of utilizing dedicated overhead relay nodes was not evaluated in our study. Future work should investigate the potential for improved transmission by using relay nodes as well as a higher density of devices. In particular, using a relay node may affect whether devices primarily transmit to one another either horizontally with cumulative vegetation effects on path loss, or vertically through forest canopy vegetation. It is important to note, however, that mesh radios used during Initial Attack on small fires may not have aircraft present to support a dedicated relay node.

There are several limitations of this study related to practical applications of our results that are important to consider. The connectivity data used in the study were compositional data that were nonnegative proportions of time that different numbers of devices were connected. The unit-sum constraint of compositional data restricts the types of analytical approaches that are appropriate. By using Dirichlet regression, our approach guaranteed that the predictions of all proportional components summed to one. One limitation to our study was the sample size (n = 24) in relation to the number of components, or connectivity levels, which was determined by the number of devices in the mesh network (six). Considerations for future work should include further evaluation of the relationship between sample size and the number of network components. In particular, a larger sample size may be necessary to investigate networks with more than six devices, as those would result in more components and thus a larger number of estimated model parameters. Because the field study was conducted using six devices in a 260-ha area, a second limitation is that results and models may not be directly transferable to either networks with different numbers of devices or to spatial extents outside of the typical PLSS section size range. Future studies should evaluate connectivity and model performance across a range of network sizes as well as across varying deployment area shapes and sizes. Finally, due in part to limited resources, logistically we could only utilize one mobile device in each section. In practice, more of the devices would most likely be mobile and future work would benefit from analyzing the connectivity of networks with different numbers of moving devices.

## Conclusions

This is the first study reporting the connectivity of smartphone-based goTenna Pro mesh networks replicated across a wide range of terrain and vegetation conditions. When deployed over PLSS sections approximately 260 ha in area, the full network of six goTenna Pros was connected on average 32.6% of the time and the mobile goTenna was disconnected from all other devices 18.2% of the time. The results show that remote sensing can successfully be used to characterize network connectivity. Vegetation affected connectivity more than topography, and the performance of these networks varied widely across sites. For example, the full network was connected 90.5% of the time in some PLSS sections but was never fully connected in other sections. Similarly, depending on the section, the mobile goTenna was not connected to any other device between 0% and 44.5% of the time. Initial Attack crews responding to wildland fires should anticipate gaps in real-time location tracking required on Type 1 fires in the United States under the Dingell Act. The Dirichlet regression models we have developed may be used to predict connectivity over large spatial extents outside of our study areas using available lidar and satellite data as predictors. One important consideration is that most of the lidar metrics used in our models were voxel-based rather than point cloud- or DEM-based. This means computation time should be considered, since voxel-based calculations tend to be more computationally complex than point cloud and DEM calculations. Ultimately, predicting connectivity in similar conditions outside the study area can be used to develop maps that forecast how well similar networks are expected to perform for wildland fire management, forestry, or other safety applications.
